# Short-term application of chicken manure under different nitrogen rates alters structure and co-occurrence pattern but not diversity of soil microbial community in wheat field

**DOI:** 10.3389/fmicb.2022.975571

**Published:** 2022-09-07

**Authors:** Haiyang Jin, Deqi Zhang, Yaqian Yan, Cheng Yang, Baoting Fang, Xiangdong Li, Yunhui Shao, Hanfang Wang, Junqin Yue, Yanjing Wang, Hongjian Cheng, Yanhua Shi, Feng Qin

**Affiliations:** ^1^Wheat Research Institute, Henan Academy of Agricultural Sciences, Zhengzhou, China; ^2^College of Agronomy, Henan Agricultural University, Zhengzhou, China

**Keywords:** manure, soil, microbial community structure, microbial community diversity, co-occurrence network, winter wheat

## Abstract

Manure application is an effective way to improve the utilization efficiency of organic resources and alleviate the adverse effects of long-term application of chemical fertilizers. However, the impact of applying manure under different nitrogen rates on soil microbial community in wheat field remains unclear. Treatments with and without chicken manure application under three nitrogen rates (N 135, 180 and 225 kg⋅hm^–2^) were set in wheat field. Soil organic carbon, available nutrients, and abundance, diversity, structure and co-occurrence pattern of soil microbial community at wheat maturity were investigated. Compared with no manure application, chicken manure application increased the soil organic carbon and available phosphorus, while the effects on soil mineral nitrogen and available potassium varied with different nitrogen rates. Chicken manure application significantly increased soil bacterial abundance under the nitrogen fertilization of 135 and 225 kg⋅hm^–2^, increased soil fungal abundance under the nitrogen fertilization of 135 kg⋅hm^–2^, but decreased soil fungal abundance under the nitrogen fertilization of 180 and 225 kg⋅hm^–2^ (*P* < 0.05). There was no significant difference in alpha diversity indices of soil microbial communities between treatments with and without chicken manure application under different nitrogen rates (*P* > 0.05). Chicken manure application and its interaction with nitrogen rate significantly changed soil bacterial and fungal community structures (*P* < 0.05). There were significantly different taxa of soil microbial communities between treatments with and without chicken manure application. Chicken manure application reduced the ecological network complexity of soil bacterial community and increased that of soil fungal community. In summary, the responses of soil available nutrients and microbial abundance to applying chicken manure varied with different nitrogen rates. One growing season application of chicken manure was sufficient to alter the soil microbial community structure, composition and co-occurrence pattern, whereas not significantly affected soil microbial community diversity.

## Introduction

For more than a century, the application of chemical nitrogen fertilizer has greatly boosted crop productivity and provided food security for about half of the population in the world (Erisman et al., 2008). Meanwhile, long-term excessive application of chemical nitrogen fertilizer has resulted in adverse consequences for crop production and environment, such as reduced fertilizer utilization efficiency (Anas et al., 2020), accelerated soil acidification (Guo et al., 2010), and increased water and air pollution (Zhang et al., 2013; Wang H. et al., 2017).

The livestock and poultry industry produces a large amount of manure with low utilization efficiency (Bai et al., 2018). However, livestock and poultry manure is an important organic resource, rich in organic matter and a variety of nutrients available for crop growth, and could be used as an effective substitution for chemical fertilizer (Du et al., 2020). Moreover, recycling manure back to cropland is also an important means to realize the organic waste reutilization and increase ecosystem service (Tang et al., 2019). Many studies have shown that application of manure (including supplemental application and partial substitution for chemical fertilizer) has multiple benefits compared to chemical fertilizer, including improving soil physical characteristics (Khaliq and Abbasi, 2015), preventing soil acidification (Wang et al., 2019), increasing soil organic carbon (Tang et al., 2019; Wang et al., 2019), increasing soil enzyme activity (Liu et al., 2021), improving soil fertility (Kobierski et al., 2017; Wei et al., 2017; Liu et al., 2021), reducing greenhouse gas emission (Tang et al., 2019), promoting crop nutrient accumulation (Xin et al., 2017; Zhang et al., 2018), improving fertilizer utilization efficiency (Xin et al., 2017; Wei et al., 2020), and increasing crop yield (Zhang et al., 2018; Du et al., 2020; Liu et al., 2021).

Soil microbial community is an important part of soil ecosystem, which is related to the regulation of nutrient biogeochemical cycling and maintenance of soil fertility. The species diversity, composition and interaction of the soil microbial community determine the resistance and stability of soil ecosystem, and are important indicators to assess soil quality (Anderson, 2003; Schloter et al., 2018). Thus, revealing the impacts of agricultural management practices on soil microbial community diversity, structure, species composition and co-occurrence pattern are vital to regulate soil ecosystem function. Among agricultural practices, fertilization (organic and chemical fertilizer) has great impact on soil microorganisms and is the primary factor affecting soil microbial community structure (Guo et al., 2020). Previous studies have shown that the application of nitrogen fertilizer and manure had significant effect on soil microbial community. Long-term nitrogen application increased the soil fungal abundance, decreased its alpha diversity, and changed its species composition (Zhou et al., 2016). Fungal community composition differed among soils under different nitrogen application rates (Zhou et al., 2016). High nitrogen rate significantly increased soil fungal abundance, while soil bacterial abundance had no significant response to different nitrogen application rates (Zhao S. et al., 2014). Compared with chemical fertilization, manure application combined with chemical fertilization could reduce the influence of environmental factors on soil microbial community (Guo et al., 2020). The application of manure increased soil microbial diversity (Legrand et al., 2018), changed the community structure and species composition of soil bacteria and fungi, had a significant impact on soil microbial ecological network and key taxa (Ji et al., 2021), and enhanced the potential soil ecosystem function (Gu et al., 2019).

The soil nutrient stoichiometry could cause changes in soil microbial community and interactions among species (Zechmeister-Boltenstern et al., 2015). The effects of nitrogen fertilization and manure application on soil microbial community in previous studies may also be related to the changes of soil resource stoichiometry. However, most studies only focused on evaluating the effects of applying chemical nitrogen or manure alone on soil microbial community, while the impact of applying manure under different nitrogen rates on soil microbial community in wheat field remains poorly understood. Consequently, this study examined the responses of chicken manure application under three nitrogen rates on soil microbial abundance, diversity, structure and ecological network. The objectives of this work were to (i) investigate the effects of chicken manure application on soil microbial abundance, diversity and structure under different nitrogen rates; and (ii) reveal the short-term effects of chicken manure application on species composition and co-occurrence pattern of soil microbial community in wheat field. We hypothesized that short-term application of chicken manure would significantly affect soil properties and microbial community, and the effect of chicken manure application would varied with different nitrogen rates, considering the change of carbon and nitrogen inputs.

## Materials and methods

### Study site

The field experiment was carried out from October 2020 to June 2021 in Haojie Village, Xigang Town, Qi County, Hebi City, Henan Province, China (35°35′ N, 114°13′ E). The average annual temperature and precipitation in this region are 14.4°C and 594 mm, respectively. The soil type was fluvo-aquic soil, and the previous crop was summer corn. The initial properties of 0–20 cm soil were as follows: organic carbon 10.84 g⋅kg^–1^, total nitrogen 1.10 g⋅kg^–1^, total phosphorus 0.38 g⋅kg^–1^, total potassium 16.37 g⋅kg^–1^, pH 8.07.

### Experimental design

In the experiment, no manure (C) and applying chicken manure (M) treatments were set under three nitrogen rates (N 135, 180 and 225 kg⋅hm^–2^), respectively. The experiment was set up in a randomized block design, with three replicates for each treatment. There were a total of 18 plots, and each plot was 7 m long and 5 m wide. The chicken manure for application was composted until thermal stabilization, and then air-dried. It contained 46.42% organic matter, 7.18% nitrogen (N), 1.82% phosphorus (P_2_O_5_), and 2.42% potassium (K_2_O). The amount of chicken manure application was 2250 kg⋅hm^–2^ dry weight. The application rates of phosphorus and potassium fertilizers in all plots were 120 kg⋅hm^–2^ P_2_O_5_ and 90 kg⋅hm^–2^ K_2_O, respectively. The 135 kg⋅hm^–2^ of nitrogen fertilizer and all of chicken manure, phosphate and potassium fertilizer were applied as basal fertilization, and the remaining nitrogen fertilizer was applied as topdressing during the jointing period of wheat. Urea (46% N), calcium superphosphate (12% P_2_O_5_) and potassium chloride (60% K_2_O) were used as chemical fertilizers. Wheat was sown on October 18, 2020. Before sowing, the basal fertilizers were evenly broadcast on the soil surface, and then the soil was rotated by tillage of 20 cm. Except for the application rate of manure and nitrogen fertilizer, the field management of each treatment was same.

### Soil sampling and chemical analysis

Soil samples were collected at the depth of 0-20 cm after harvesting the winter wheat on June 5, 2021. Five soil cores (2.5 cm diameter) were collected randomly from each plot and mixed as soil sample for the plot. Soil samples were sieved through 5 mm to remove impurities and further homogenize. Each sample was divided into two parts and brought back to the laboratory on ice. One was stored at a −80°C for soil microbial community analysis, and the other was air-dried for soil organic carbon and available nutrient analysis.

Soil organic carbon (SOC) was determined by potassium dichromate volumetric method. Soil NO_3_^–^-N and NH_4_^+^-N were determined by continuous flow analyzer after extracting with 1 mol⋅L^–1^ KCl. Soil mineral nitrogen was expressed as the total concentration of NO_3_^–^-N and NH_4_^+^-N. Soil available phosphorus was extracted with 0.5 mol⋅L^–1^ NaHCO_3_ and determined by the molybdenum antimony colorimetric method. Soil available potassium was extracted with 1 mol⋅L^–1^ NH_4_OAc and determined by flame photometry.

### DNA extraction and real-time fluorescent quantitative polymerase chain reaction

Soil total DNA was extracted from ∼0.5 g of each soil sample using the FastDNA Spin Kit for Soil (MP Biomedicals, United States). The concentration and quality of the extracted DNA were evaluated using NanoDrop 2000 (Thermo Scientific, United States) and 1% agarose gel electrophoresis.

Real-time fluorescent quantitative polymerase chain reaction (PCR) of the bacterial 16S rRNA gene and the fungal ITS sequence was performed using the primers 515F/907R (Song et al., 2019) and ITS3F/ITS4R (Gade et al., 2013). The qPCR reagent kit was ChamQ SYBR Color qPCR Master Mix (Cat. No. Q411-02, Vazyme Biotech, China). According to the pre-experiment results, the optimal annealing temperatures for the 16S rRNA gene and ITS sequence were set at 60°C and 58°C, respectively. A total of 40 cycles of qPCR were performed. The plasmid DNA containing the target sequence with known concentration was used as standard. The standard series was prepared by a 10-fold serial dilution, which was detected by qPCR at the same time as the sample. Each sample and the standard were measured three times to obtain the average value, and the copy number of the target sequence in the sample was calculated according to the standard curve.

### PCR amplification and high-throughput sequencing

PCR amplification of the bacterial 16S rRNA gene and the fungal ITS sequence was performed using the primers 515F/907R (Song et al., 2019) and ITS3F/ITS4R (Gade et al., 2013). The PCR reagent kit was TransStart FastPfu DNA Polymerase (CAT. No. Ap221-02, TransGen Biotech, China). PCR conditions for the bacterial 16S rRNA gene and fungal ITS sequence were as follows: 3 min of initial denaturation at 95°C, followed by 27 (16S rRNA gene) and 35 (ITS sequence) cycles of 95°C for 30 s, 55°C for 30 s and 72°C for 45 s, and then a final extension step at 72°C for 10 min. The PCR amplification volume was 20 μL, and each sample had three replicate wells. The AxyPrep DNA Gel Extraction Kit (CAT. No. AP-GX-250, Axygen, United States) was used to obtain the target DNA fragment. Miseq PE300 high-throughput sequencing was performed after the sequencing library preparation.

### Statistical analysis

The high-throughput sequencing data were analyzed using the online platform of Majorbio Cloud Platform^1^ (Ren et al., 2022). FLASH (Magoč and Salzberg, 2011) and fastp (Chen et al., 2018) were used for assembly, quality control and filtering of the raw data. USEARCH (Edgar, 2010) was used for OTU clustering according to 97% similarity, and the sequences were randomly resampled with the minimum sequence number. The OTU taxonomy assignment of bacteria and fungi was performed by RDP Classifier (Wang et al., 2007) based on the SILVA (Pruesse et al., 2007) and UNITE (Nilsson et al., 2019) databases, respectively. The mothur (Schloss, 2020) was used to calculate alpha diversity indices (Chao1 index and Shannon index) of soil microbial community at OTU level. Non-metric multidimensional scale analysis (NMDS) based on Abund Jaccard distance algorithm and similarity analysis (ANOSIM) were used to analyze the differences of soil microbial community structure. Mantel test was used to analyze the correlation between soil microbial community structure and soil properties. Linear discriminant analysis effect size (LEfSe) analysis (LDA > 3) (Segata et al., 2011) was used to analyze the differential taxa of soil microbial communities in treatments with and without chicken manure application. According to the Spearman correlation (| r | > 0.7, Benjamini-Hochberg adjusted *P* < 0.05), soil microbial ecological network analysis among genera with relative abundance of >0.1% was performed by the Cytoscape plugin CoNet (Faust and Raes, 2016). Topological parameter of the network was calculated by NetworkAnalyzer (Assenov et al., 2008). Finally, Gephi was used to visualize the network. Soil organic carbon, available nutrients, microbial abundance and alpha diversity indices among different treatments were compared under ANOVA and LSD test for multiple comparisons at *P* < 0.05 in SPSS.

## Results

### Soil organic carbon and available nutrients

**FIGURE 1:**
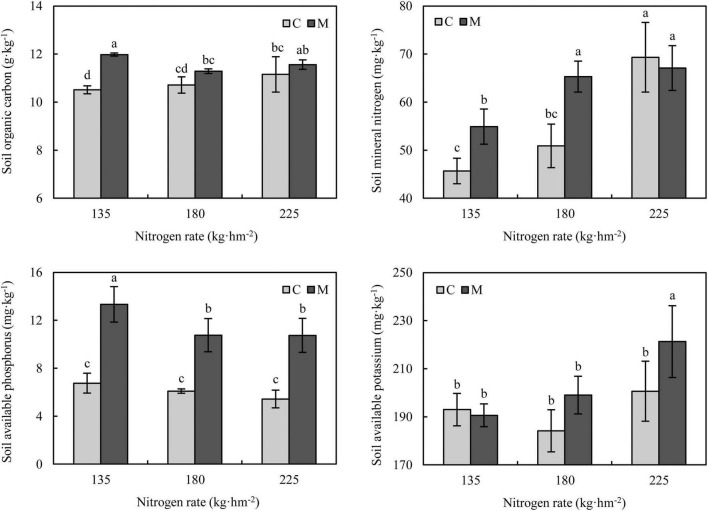
Soil organic carbon and available nutrients under different nitrogen and manure treatments. M and C indicate treatments with and without chicken manure application, respectively. Error bars represent standard deviations (*n* = 3). Different letters above bars indicate significant difference at 0.05 level.

Soil organic carbon and available nutrients under different treatments are shown in Figure 1. Compared with no manure application, manure application increased soil organic carbon, and the increase rate was the largest under 135 kg⋅hm^–2^ nitrogen fertilization (increased by 13.90%). With the increase of nitrogen rate, the soil mineral nitrogen was increased at wheat maturity. Manure application significantly increased soil mineral nitrogen under 135 and 180 kg⋅hm^–2^ nitrogen fertilization (*P* < 0.05), while there was no significant difference in soil mineral nitrogen between treatments with and without manure application under 225 kg⋅hm^–2^ nitrogen fertilization (*P* > 0.05). Compared with no manure application, applying manure significantly increased soil available phosphorus under different nitrogen rates (*P* < 0.05), the increase rates were 76.71–97.87%. The effect of manure application on soil available potassium was not significant under 135 and 180 kg⋅hm^–2^ nitrogen fertilization (*P* > 0.05), while manure application under 225 kg⋅hm^–2^ nitrogen rate significantly increased soil available potassium (*P* < 0.05).

### Soil microbial abundance

**FIGURE 2:**
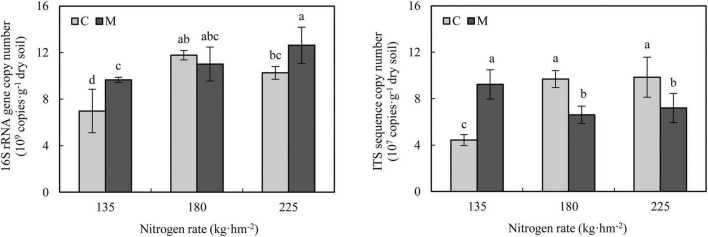
Soil microbial abundance under different nitrogen and manure treatments. M and C indicate treatments with and without chicken manure application, respectively. Error bars represent standard deviations (*n* = 3). Different letters above bars indicate significant difference at 0.05 level.

Quantitative PCR analysis showed that compared with 135 kg⋅hm^–2^ nitrogen fertilization, increasing nitrogen rate (180 and 225 kg⋅hm^–2^) increased the abundance of soil bacteria (Figure 2). Under 135 and 225 kg⋅hm^–2^ nitrogen fertilization, the application of manure significantly increased the abundance of soil bacteria (*P* < 0.05). Compared with 135 kg⋅hm^–2^ nitrogen fertilization, increasing nitrogen rate (180 and 225 kg⋅hm^–2^) without manure application significantly increased the abundance of soil fungi (*P* < 0.05), while increasing nitrogen fertilizer rate (180 and 225 kg⋅hm^–2^) with manure application significantly decreased the abundance of soil fungi (*P* < 0.05), which indicated that application of manure changed the response of soil fungal abundance to nitrogen rate. Compared with no manure application, the application of manure significantly increased the soil fungal abundance under 135 kg⋅hm^–2^ nitrogen fertilization (*P* < 0.05), but significantly decreased soil fungal abundance under 180 and 225 kg⋅hm^–2^ nitrogen fertilization (*P* < 0.05).

### Alpha diversity and structure of soil microbial community

After the sequencing data of 16S rRNA gene were randomly resampled according to the minimum sequence number, 27952 sequences with an average length of 377 bp were obtained for each sample. After OTU taxonomy assignment, a total of 4860 OTUs were obtained from 18 samples, belonging to 828 genera, 471 families, 298 orders, 125 classes and 45 phyla. After the sequencing data of ITS sequence were randomly resampled according to the minimum sequence number, 49288 sequences with an average length of 312 bp were extracted from each sample. After OTU taxonomy assignment, a total of 1400 OTUs were obtained from 18 samples, belonging to 394 genera, 209 families and 104 orders, 53 classes and 18 phyla.

The alpha diversity indices (Chao1 richness index and Shannon diversity index) of soil microbial community in different treatments are shown in Table 1. Under the same nitrogen rate, there was no significant difference in alpha diversity indices of soil microbial communities between treatments with and without manure application (*P* > 0.05). In addition, there was no significant difference in alpha diversity indices of soil microbial communities among treatments with manure application under different nitrogen rates (*P* > 0.05). The above results indicated that short-term nitrogen rate and manure application had little impact on the alpha diversity of soil microbial community.

**TABLE 1:** Soil microbial community alpha diversity indices under different nitrogen and manure treatments.

Treatment	Bacterial community		Fungal community	
		
	Chao1 index	Shannon index	Chao1 index	Shannon index
C135	3451 ± 161ab	6.60 ± 0.04a	614 ± 54a	4.16 ± 0.28a
M135	3296 ± 37b	6.57 ± 0.01ab	641 ± 92a	3.63 ± 0.85ab
C180	3484 ± 103a	6.52 ± 0.03b	582 ± 51a	3.91 ± 0.49ab
M180	3401 ± 31ab	6.53 ± 0.04b	606 ± 20a	3.94 ± 0.04ab
C225	3326 ± 101ab	6.59 ± 0.01a	617 ± 57a	3.96 ± 0.41ab
M225	3342 ± 111ab	6.57 ± 0.03ab	591 ± 78a	3.12 ± 0.72b

Values are means ± standard deviation (n = 3). Different letters in the same column indicate significant difference at 0.05 level. C, no manure; M, applying manure.

**FIGURE 3:**
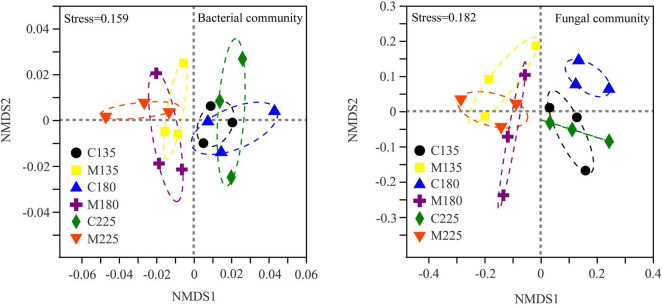
Non-metric multidimensional scaling analysis of soil microbial communities under different nitrogen and manure treatments. M and C indicate treatments with and without chicken manure application, respectively.

As shown in Figure 3, non-metric multidimensional scale analysis (NMDS) was used to study the differences of soil microbial community structure under different treatments. The structure of soil bacterial and fungal community responded identically to different treatments. The soil microbial communities in treatments with and without manure application were separated obviously at NMDS1 axis, while there was no obvious separation among different nitrogen rates. The results of similarity analysis (ANOSIM) were shown in Table 2. Nitrogen rate had no significant effect on soil bacterial and fungal community structure, while manure application and its interaction with nitrogen rate significantly changed soil bacterial and fungal community structure (*P* < 0.05).

**TABLE 2:** Similarity analysis (ANOSIM) of soil microbial communities under different nitrogen and manure treatments.

Characteristics	Bacterial community		Fungal community	
		
	*r*	*P*-value	*r*	*P*-value
Nitrogen rate	−0.0374	0.661	−0.0033	0.456
Manure application	0.5175	0.001	0.6235	0.001
Nitrogen rate × Manure application	0.2354	0.024	0.4058	0.001

Since the application of manure had the significant effect on soil microbial community structure, Linear discriminant analysis effect size (LEfSe) analysis was further used to recognize the differential taxa between treatments with and without manure application (LDA > 3). A total of 13 bacterial taxa and 58 fungal taxa were identified with significant differences (Supplementary Table 1). In soil bacterial community, the family *Comamonadaceae* was significantly enriched in no manure application treatments, while the order *Solirubrobacterales* and *Gaiellales*, the family *Entotheonellaceae*, and the genus *Luteimonas* were significantly enriched in manure application treatments. In soil fungal community, the treatments without manure application had significant enrichment of 14 genera, 8 families, 5 orders and 2 classes, and treatments with manure application had significant enrichment of 16 genera, 9 families, 3 orders and 1 class. At the genus level, *Fusariella*, unclassified *Sympoventuriaceae, Preussia, Neosetophoma, Alternaria, Rhizophlyctis, Ochroconis, Schizothecium*, unclassified *Dothideomycetes, Setophoma, Monocillium*, unclassified *Sordariomycetes, Podospora* and *Staphylotrichum* were significantly enriched in the treatments without manure application, while *Leucothecium, Niesslia, Actinomucor*, unclassified *Ascodesmidaceae, Diutina, Cephaliophora, Dactylaria, Pseudaleuria, Cutaneotrichosporon, Microascus, Lophotrichus*, unclassified *Sporormiaceae, Neonectria*, unclassified *Chaetomiaceae, Hyalorbilia* and *Thelonectria* were significantly enriched in the treatments with manure application.

### Correlations between soil microbial community structure and soil properties

As shown in Table 3, mantel test was conducted to analyze the correlation between soil microbial community structure and soil properties. Soil bacterial and fungal community structures were significantly correlated with soil available phosphorus and potassium (*P* < 0.05), and soil fungal community structure was also significantly correlated with soil organic carbon (*P* < 0.05), while the correlation between soil microbial community structure and soil mineral nitrogen was not significant (*P* > 0.05).

**TABLE 3:** Mantel test correlations between soil microbial community structure and soil properties.

Soil property	Bacterial community		Fungal community	
		
	*r*	*P*-value	*r*	*P*-value
Soil organic carbon	0.0498	0.595	0.2817	0.011
Soil mineral nitrogen	−0.0574	0.622	0.0261	0.801
Soil available phosphorus	0.3215	0.002	0.4817	0.001
Soil available potassium	0.4590	0.002	0.3418	0.011

### Network analysis of soil microbial community

**FIGURE 4:**
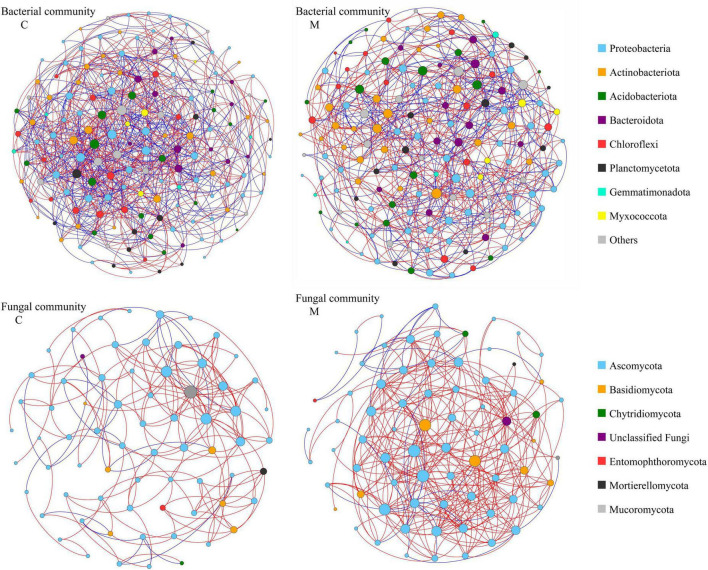
Ecological networks of soil bacterial community and fungal community under different organic manure treatments. M and C indicate treatments with and without chicken manure application, respectively. Genera are represented as nodes and correlations as edges (red: copresence, blue: mutual exclusion). The node sizes are correlated to the genus degree, and node color indicates the corresponding taxonomic assignment at phylum level.

In order to decipher the potential interactions among microbial taxa, network analysis was performed with sequence data of soil microbial community. As shown in Figure 4 and Table 4, the soil bacterial ecological network of no manure application treatments was composed of 160 nodes and 967 edges, and the soil bacterial ecological network of manure application treatments was composed of 161 nodes and 710 edges. The average number of neighbors, clustering coefficient and network density of soil bacterial ecological network of manure application treatments were smaller than those of no manure application treatments, which indicated that applying manure reduced the complexity of soil bacterial ecological network. The response of soil fungal ecological network to manure application was on the contrary to that of soil bacterial community. The soil fungal ecological network of no manure application treatments was composed of 70 nodes and 186 edges, and the soil fungal ecological network of manure application treatments was composed of 74 nodes and 356 edges. Compared with no manure application, the average number of neighbors, clustering coefficient and network density of soil fungal ecological network were increased by applying manure, which indicated that applying manure increased the complexity of soil fungal ecological network. For the ecological networks of soil total microbial community, compared with no manure application, manure application decreased the number of total and mutual exclusion edges, while there was little difference of other topological parameters (Supplementary Figure 1 and Table 4). As shown in Supplementary Table 2, the percentage of bacterial-bacterial correlation was higher than those of fungal-fungal and bacterial-fungal correlations in soil total microbial networks. Compared with no manure application, manure application decreased the percentage of bacterial-bacterial correlation and increased the percentage of fungal-fungal correlation in soil total microbial network.

**TABLE 4:** Topological parameters of soil microbial ecological networks under different organic manure treatments.

Topological parameter	Bacterial community		Fungal community		Total microbial community	
			
	C	M	C	M	C	M
Number of nodes	160	161	70	74	236	237
Number of edges	967	710	186	356	1643	1523
Number of copresence edges	496	380	158	321	932	954
Number of mutual exclusion edges	471	330	28	35	711	569
Average number of neighbors	12.088	8.820	5.314	9.944	13.924	12.852
Clustering coefficient	0.388	0.364	0.380	0.466	0.369	0.349
Network density	0.076	0.055	0.077	0.142	0.059	0.054

C, no manure; M, applying manure.

## Discussion

### Effects of manure application under different nitrogen rates on soil organic carbon and available nutrients

It is expected that soil organic carbon and available nutrients could be affected by manure application because manure itself is rich in organic carbon and available nutrients. In this study, manure application increased soil organic carbon under different nitrogen rates (Figure 1), which was consistent with the results of previous studies (Zhong et al., 2010; Wei et al., 2017; Lin et al., 2019). The increase of soil organic carbon was related to the additional carbon input. Although the increase in soil carbon input could stimulate the mineralization of soil organic carbon, the replenishment was more than priming loss of soil organic carbon with additional carbon input (Liang et al., 2018). The results of previous studies showed that the soil available phosphorus with long-term manure application was significantly higher than that without (Zhong et al., 2010; Kobierski et al., 2017; Wei et al., 2017). Ma et al. found that short-term application of manure (four rice growing seasons) significantly enriched soil total and available phosphorus (Ma et al., 2016). In this study, applying chicken manure in one wheat growing season could significantly increase the soil available phosphorus at wheat maturity, with an increase of 76.71-97.87% (Figure 1). It has been demonstrated that soil available phosphorus was significantly positively correlated with soil organic matter (Shen et al., 2014). Compared with chemical fertilization, manure application could improve the activity of soil phosphatase, which is responsible for soil phosphorus cycling and could increase available phosphorus (Li et al., 2015). The improvement of soil phosphatase activity by manure application may be another reason for the increase of soil available phosphorus in this study. Therefore, it may be feasible to rapidly increase soil available phosphorus by applying chicken manure to meet the phosphorus demand of crop production in phosphorus-restricted soil. Meanwhile, it could not be ignored that a large amount of available phosphorus residues at crop harvest may increase the risk of phosphorus leaching.

The results also showed that the responses of soil organic carbon, mineral nitrogen and available potassium to manure application were affected by different nitrogen rates (Figure 1). The increase of soil organic carbon and mineral nitrogen with manure application under low nitrogen rate was greater than that under high nitrogen rate, and the difference was not significant under high nitrogen rate. The response of soil available potassium was on the contrary that the application of manure under low nitrogen rate had no significant effect on soil available potassium, while increased significantly under high nitrogen rate. It was speculated that these results may be related to crop absorption under different treatments and soil enzyme activity under different soil nutrient stoichiometry.

### Effects of manure application under different nitrogen rates on soil microbial community diversity and structure

This short-term experimental results showed that there was no significant difference in alpha diversity indices of soil microbial communities among treatments with manure application under different nitrogen rates (*P* > 0.05) (Table 1). In previous studies, the effects of manure application on soil microbial alpha diversity were inconsistent among different crop systems. In a double-cropping rice system, 3-year manure application increased soil bacterial and fungal alpha diversity compared to chemical fertilization (Tang et al., 2020). In a broccoli system, soil bacterial community richness index was significantly increased by short-term application of chicken manure, but there was no significant difference in soil bacterial Shannon diversity index between chicken manure application and control soil (Ye et al., 2022). In a winter wheat-summer maize system, there was no significant difference in Chao1 and Shannon indices of soil bacterial and fungal community between short-term manure and chemical fertilizer treatments (Wang et al., 2022). In a rice-wheat rotation system, long-term manure application had no significant effect on Shannon index of soil bacterial and fungal community, and Chao1 index of soil fungal community, while significantly increased the Chao1 index of soil bacterial community (Wang J. et al., 2017). The results of this and previous studies showed that soil microbial community was acclimated to manure application, and the reasons for the inconsistent results in different experiments may be related to the soil and manure types.

Wang et al. found that fertilization regime of wheat and rice did not change the soil bacterial community diversity, but changed the soil bacterial community structure (Wang et al., 2016). Similarly, the results showed that the application of manure had no significant effect on the diversity, but significantly changed the structure of soil microbial community (Figure 3), which indicated that soil microbial community structure was more sensitive than soil microbial community diversity to the application of manure. Our results echoed the viewpoint of previous studies what the soil bacterial and fungal community structure could be significantly affected by manure application (Gu et al., 2019; Lin et al., 2019; Ren et al., 2021). In line with the previous research (Wei et al., 2017; Luan et al., 2020), the results of NMDS and ANOSIM showed that the impact of manure application on soil microbial community structure was more significant than that of nitrogen rate, and manure application and its interaction with nitrogen rate significantly changed soil bacterial and fungal community structure (*P* < 0.05) (Figure 3 and Table 2). It was suggested that the mineralization of organic matter in manure is a long-term process, which is difficult to fully exert its nutritional potential on soil microbial community in a short-term period (Zhang M. et al., 2021). Chemical nitrogen fertilizers had stronger effect on soil microbial community than manure under short-term condition, as nitrogen addition could increase microbial activities by mitigating nitrogen limitation (Zhang M. et al., 2021). While in this study, nitrogen may not be the main factor limiting soil microbial growth. Mantel test results further demonstrated that soil microbial community structure was not significantly correlated with soil mineral nitrogen, but significantly correlated with soil available phosphorus and potassium (Table 3). The results were supported by Ma et al. who found that phosphorus and phosphorus-related nutrient stoichiometry drove the changes of soil microbial community composition in paddy soil (Ma et al., 2016). Additionally, livestock and poultry manure is rich in gut microbial communities (Bebber and Richards, 2022), and the microorganisms in manure may also have an impact on the structure of soil microbial community. Studies have shown that gut microorganisms could survive in manure- or compost- amended soils for a long time (Sharma and Reynnells, 2016), and diverse fungal taxa in manure were transmitted into soil by the application of manure, which account for 1.10-2.04% of the relative abundance of soil fungal community (Sun et al., 2020). Therefore, the effect of manure application on soil microbial community structure could also be attributed to the microbial community brought by manure itself.

This study used the same LDA threshold for soil bacterial and fungal communities to detect the differential taxa between treatments with and without manure application. The results showed that the number of differential taxa of fungi was more than that of bacteria (Supplementary Table 1), which indicated that the effect of manure application on composition of fungal community was greater than that of bacterial community. For soil bacterial community, manure application reduced relative abundance of the family *Comamonadaceae*. Certain members of the family *Comamonadaceae* have been reported to exhibit extensive metabolic pathways, such as degradation of cyclohexane, accumulation of polyphosphate, denitrification, etc. (Ding et al., 2019). Previous results confirmed that exogenous fertilizers significantly affected an unclassified *Comamonadaceae*, and the unclassified *Comamonadaceae* was negatively correlated with soil nitrogen mineralization (Wu et al., 2021). The results also showed that the order *Gaiellales* and genus *Luteimonas* were significantly enriched in manure application treatments. In a field experiment with Ultisols, *Gaiellales* was also found to be enriched by pig manure application (Lin et al., 2019). *Luteimonas* is a *nirK*-type denitrifier, which is associated with soil nitrogen loss and nitrous oxide emission (Zhong et al., 2020). For soil fungal community, manure application reduced the relative abundance of potential pathogens, such as *Alternari*a (Lou et al., 2013), *Setophoma* (Ikeda et al., 2012; Yoshida, 2022), unclassified *Sordariomycetes* (Maharachchikumbura et al., 2015, 2016). Among the genus-level taxa enriched by manure application, *Dactylaria* and *Cutaneotrichosporon* were potentially beneficial fungi. In previous studies, *Dactylaria brochopaga* of the genus *Dactylaria* could manage root-knot disease of wheat by trapping *Meloidogyne graminicola* (Kumar and Singh, 2011). *Dactylaria higginsii* of the genus *Dactylaria* could be used as fungal bioherbicide agent for purple nutsedge (Kadir and Charudattan, 2000). *Cutaneotrichosporon* was negatively associated with *Fusarium oxysporum*, and could inhibit the infection of *Fusarium oxysporum* (Zhu et al., 2022). Notably, manure application also enriched coprophilous fungi such as *Niesslia*, *Cephaliophora*, *Microascus* and *Chaetomiaceae* (Dischler et al., 2019; Zhang X. et al., 2021). Due to the lack of sufficient culture studies for some differential species, it was difficult to estimate the ecological impact brought about by the changes in their relative abundance. It could be confirmed that the differences in soil microbial community composition caused by manure application would further affect soil ecological function. On the other hand, the different composition of soil microbial community may also cause changes in the interactions among soil microbial taxa.

### Effects of manure application on soil microbial ecological network

Various microbial taxa do not exist independently in ecosystem, but interact with each other to form a complex network (Zhou et al., 2010). The co-occurrence network analysis could explore the interactions among soil microbial taxa (Zhang M. et al., 2021), which play an important role in ecosystem process and function. In this study, the response of co-occurrence network to manure application was different for bacterial and fungal communities (Figure 4 and Table 4). After one growing season, applying manure decreased the average number of neighbors, clustering coefficient and network density of soil bacterial ecological network, but increased those of soil fungal ecological network, which implied that manure application decreased the complexity of soil bacterial ecological network and increased the complexity of soil fungal ecological network. The previous study has shown that the effect of manure application on soil bacterial network was related to the application rate, that is, low manure application rate could lead to complex and stable bacterial community, while high manure application rate decreased the stability of bacterial network (Liu et al., 2020). For soil fungal community, previous studies have consistently shown that manure application could increase the network edges, average degree and density (Wang et al., 2022), and enhance the network complexity (Ji et al., 2020). As the C/N ratio of fungal hyphae is higher than that of bacterial cells (Wallander et al., 2003), bacteria and fungi often exhibit different substrate preference. Manure application increased soil carbon input and the C/N ratio, which was beneficial to the growth of soil fungi (Grosso et al., 2016). Therefore, it was reasonable that manure application increased the interaction between soil fungal species. The topological data indicated that the increase of edge number in soil fungal network was mainly due to the change of positive links (Table 4). Positive correlations between microorganisms in ecological network could be explained by common preferred conditions or cooperative activities (Fuhrman, 2009). It could be speculated that the recalcitrant organic matters introduced by manure application required more microbial cooperation to be degraded, which resulted in a complex fungal ecological network (Ye et al., 2021). The more complex network indicates that there is a closer interaction among microorganisms, which would be beneficial to resist the environmental stresses (Ling et al., 2016). In this study, manure application decreased the number of mutual exclusion edges in the ecological network of soil total microbial community. The studies of ecological theory showed that competitive interactions could promote stability of ecological networks (Coyte et al., 2015). It is worth noting that interspecific interactions within microbial community are highly complex, further studies with more experimental evidence are needed to compensate for the limitations of co-occurrence network analysis only (Banerjee et al., 2018; Guseva et al., 2022; Jin et al., 2022).

In general, this study showed that application of chicken manure could alter soil properties, microbial community structure, species composition and co-occurrence pattern in a short-term period. At the same time, it should be noted that manure is also a pollution source rich in heavy metals, antibiotics and antibiotic resistance genes (Zhao Y. et al., 2014; Guo et al., 2018), and the potential environmental risks of long-term manure application under the condition of this study need to be further evaluated.

## Conclusion

Results in this study showed that one growing season application of chicken manure significantly increased soil available phosphorus under different nitrogen rates, significantly increased soil organic carbon and mineral nitrogen under low nitrogen rate, and significantly increased soil available potassium under high nitrogen rate. The response of soil bacterial and fungal abundance to applying chicken manure varied with different nitrogen rates. Chicken manure application and nitrogen rate did not significantly affect soil microbial community alpha diversity in a short term, while applying chicken manure significantly changed soil microbial community structure and species composition. The application of chicken manure reduced the complexity of soil bacterial ecological network and increased the complexity of soil fungal ecological network.

## Data availability statement

The datasets presented in this study can be found in online repositories. The names of the repository/repositories and accession number(s) can be found below: https://www.ncbi.nlm.nih.gov/, PRJNA851220.

## Author contributions

HJ, DZ, and XL designed the work. HJ, DZ, and YY analyzed the data and wrote the manuscript. CY, BF, and JY performed laboratory measurements. HC and YaS carried out field sampling. YuS, HW, and FQ performed the field experiment. YW and XL revised the manuscript. All authors discussed the results and approved the final manuscript.

## Footnotes

1
https://cloud.majorbio.com/
