# Supplementary material for: Short-term application of chicken manure under different nitrogen rates alters structure and co-occurrence pattern but not diversity of soil microbial community in wheat field
Source: Front Microbiol. 2022 Sep 7;13:975571. doi: 10.3389/fmicb.2022.975571 (PMC9490364; doi:10.3389/fmicb.2022.975571)
Supplement: Supplementary file 1 [file Data_Sheet_1.DOCX]

Supplementary Material

# Supplementary Figures and Tables

## Supplementary Figures

**Supplementary Figure 1** Ecological networks of soil total microbial community under different organic manure treatments. M and C indicate treatments with and without chicken manure application, respectively. Genera are represented as nodes and correlations as edges (red: copresence, blue: mutual exclusion). The node sizes are correlated to the genus degree, and node colour indicates the corresponding taxonomic assignment at phylum level.

## Supplementary Tables

**Supplementary Table 1** Differential taxa of soil microbial communities under different manure treatments.

| Kingdom | Taxa name | Enriched group | LDA value |
| --- | --- | --- | --- |
| Bacteria | f_Comamonadaceae | C | 3.37 |
| Bacteria | o_Burkholderiales | C | 3.63 |
| Bacteria | p_Entotheonellaeota | M | 3.14 |
| Bacteria | o_Solirubrobacterales | M | 3.07 |
| Bacteria | o_Entotheonellales | M | 3.14 |
| Bacteria | f_Entotheonellaceae | M | 3.14 |
| Bacteria | g_norank f_Entotheonellaceae | M | 3.14 |
| Bacteria | c_Entotheonellia | M | 3.14 |
| Bacteria | c_Thermoleophilia | M | 3.52 |
| Bacteria | g_Luteimonas | M | 3.03 |
| Bacteria | o_Gaiellales | M | 3.29 |
| Bacteria | g_norank f_67-14 | M | 3.06 |
| Bacteria | f_67-14 | M | 3.06 |
| Fungi | c_Rhizophlyctidomycetes | C | 3.55 |
| Fungi | f_Lasiosphaeriaceae | C | 4.73 |
| Fungi | f_Sporormiaceae | C | 4.37 |
| Fungi | f_Pleosporaceae | C | 3.72 |
| Fungi | o_Pleosporales | C | 4.77 |
| Fungi | o_Venturiales | C | 3.35 |
| Fungi | g_Fusariella | C | 3.04 |
| Fungi | g_unclassified f_Sympoventuriaceae | C | 3.92 |
| Fungi | g_Preussia | C | 4.37 |
| Fungi | g_Neosetophoma | C | 3.18 |
| Fungi | g_Alternaria | C | 3.74 |
| Fungi | g_Rhizophlyctis | C | 3.55 |
| Fungi | g_Ochroconis | C | 3.68 |
| Fungi | f_Rhizophlyctidaceae | C | 3.55 |
| Fungi | g_Schizothecium | C | 4.16 |
| Fungi | f_Sympoventuriaceae | C | 3.35 |
| Fungi | o_unclassified c_Dothideomycetes | C | 3.57 |
| Fungi | g_unclassified c_Dothideomycetes | C | 3.57 |
| Fungi | g_Setophoma | C | 3.16 |
| Fungi | o_Rhizophlyctidales | C | 3.55 |
| Fungi | g_Monocillium | C | 3.14 |
| Fungi | g_unclassified c_Sordariomycetes | C | 3.13 |
| Fungi | g_Podospora | C | 4.55 |
| Fungi | c_Dothideomycetes | C | 4.83 |
| Fungi | g_Staphylotrichum | C | 3.45 |
| Fungi | f_unclassified c_Dothideomycetes | C | 3.57 |
| Fungi | o_unclassified c_Sordariomycetes | C | 3.13 |
| Fungi | f_unclassified c_Sordariomycetes | C | 3.13 |
| Fungi | f_Sordariales fam Incertae sedis | C | 3.47 |
| Fungi | o_Pezizales | M | 4.84 |
| Fungi | g_Leucothecium | M | 3.54 |
| Fungi | g_Niesslia | M | 3.62 |
| Fungi | o_Microascales | M | 3.62 |
| Fungi | c_Pezizomycetes | M | 4.84 |
| Fungi | g_Actinomucor | M | 3.02 |
| Fungi | g_unclassified f_Ascodesmidaceae | M | 3.93 |
| Fungi | f_Saccharomycetales fam Incertae sedis | M | 3.12 |
| Fungi | g_Diutina | M | 3.02 |
| Fungi | g_Cephaliophora | M | 4.81 |
| Fungi | f_Niessliaceae | M | 3.62 |
| Fungi | g_Dactylaria | M | 4.15 |
| Fungi | g_Pseudaleuria | M | 3.52 |
| Fungi | f_Pyronemataceae | M | 3.62 |
| Fungi | g_Cutaneotrichosporon | M | 3.06 |
| Fungi | f_Thelebolaceae | M | 3.70 |
| Fungi | f_Microascaceae | M | 3.62 |
| Fungi | f_Chaetomiaceae | M | 4.91 |
| Fungi | f_Trichosporonaceae | M | 3.22 |
| Fungi | g_Microascus | M | 3.36 |
| Fungi | f_Mucoraceae | M | 3.04 |
| Fungi | g_Lophotrichus | M | 3.57 |
| Fungi | f_Ascodesmidaceae | M | 4.81 |
| Fungi | g_unclassified f_Sporormiaceae | M | 3.28 |
| Fungi | g_Neonectria | M | 3.40 |
| Fungi | g_unclassified f_Chaetomiaceae | M | 4.93 |
| Fungi | o_Trichosporonales | M | 3.22 |
| Fungi | g_Hyalorbilia | M | 3.00 |
| Fungi | g_Thelonectria | M | 3.21 |

C, no manure; M, applying manure

**Supplementary Table 2** The percentages of bacterial-bacterial, fungal-fungal and bacterial-fungal correlations in soil total microbial network.

| Correlation | No manure (C) | | |  | Applying manure (M) | | |
| --- | --- | --- | --- | --- | --- | --- | --- |
|  | Bacterial-Bacterial | Fungal-Fungal | Bacterial-Fungal |  | Bacterial-Bacterial | Fungal-Fungal | Bacterial-Fungal |
| Copresence (%) | 53.22 | 16.95 | 29.83 |  | 39.83 | 33.65 | 26.52 |
| Mutual exclusion (%) | 66.24 | 3.94 | 29.82 |  | 58.00 | 6.15 | 35.85 |
